# Fishing for elusive cCMP-degrading phosphodiesterases

**DOI:** 10.1186/2050-6511-14-S1-P62

**Published:** 2013-08-29

**Authors:** Erich Schneider, Maike Kuhn, Daniel Reinecke, Sabine Wolter, Heike Burhenne, Volkhard Kaever, Roland Seifert

**Affiliations:** 1 Institute of Pharmacology, Hannover Medical School (MHH), Germany; 2 Core Unit Mass Spectrometry & Metabolomics, MHH, Germany

## Background

The literature of the past four decades contains several scattered reports that demonstrate the existence of two different 3’,5’-cCMP degrading enzymes with distinct substrate affinity and specificity. The so-called “cCMP-specific” PDE was isolated from disrupted L-1210 leukemia cells [1] and from rat liver [2,3], shows an extremely high 3’,5’-cCMP K_M_ value of up to 9 mM and seems to be activated by iron [1]. This enzyme prefers 3’,5’-cCMP over other cyclic nucleotides. The other enzyme is called “multifunctional cCMP PDE” and was isolated from pig liver [3-5], rat liver [6,7] and human liver [8]. This enzyme shows a 3’,5’-cCMP K_M_ value in the range of 100-300 µM and is additionally degrading 2’,3’-cCMP as well as the 2’,3’- and 3’,5’-isomers of cUMP, cAMP, cGMP and cIMP. The multifunctional cCMP PDE is inhibited by inorganic phosphate and AMP. Both enzymes are calmodulin-insensitive, IBMX-resistant and show a low molecular weight of <35 kDa, which is uncommon for the “classic” phosphodiesterases. However, the exact amino acid sequence and identity of these proteins remain elusive.

Our project aims at a clear and unequivocal determination of amino acid sequence and identity of these elusive cCMP-degrading enzymes by analyzing and purifying the cCMP degrading activity of organ extracts and biological fluids (e.g. serum). Moreover, we re-investigate well-known “classic” PDEs in enzymatic assays to identify a potential 3’,5’-cCMP degrading activity.

## Materials and methods

The substrates and products of PDE reactions are detected and quantified with high accuracy by a highly sensitive HPLC-MS/MS method (triple quadrupol mass spectrometer) which is established in our institute. This method enables us for the first time to unequivocally identify cyclic nucleotides and their degradation products in biological samples.

We assay organ extracts and biological fluids for cCMP PDE activity and use the standard methods of protein biochemistry to identify, concentrate and purify these enzymes (Western blot, SDS PAGE, fractionated precipitation of proteins by addition of ammonium sulfate or lowering pH value). A very important part of our methodology is the use of an FPLC system for protein separation. The identity of purified proteins will be determined by analyzing a fingerprint of the fragments in mass spectrometry and comparing the results with the content of biological databases. In addition, we characterize the enzymatic activity of commercially available recombinant PDEs.

## Results

A screen of 13 commercially available recombinant PDEs revealed that these enzymes show unexpectedly broad substrate specificity and also degrade “exotic” cyclic nucleotides like cUMP, cIMP, cXMP and cTMP [9]. Most interestingly, however, only one of the tested enzymes, PDE 7A1, degraded cCMP.

In addition, we have identified a heat-sensitive cCMP-degrading activity in certain commercially available batches of fetal bovine serum. This enzymatic activity can be considerably enhanced by filtration of the serum through centrifugation filtration devices (30 kDa cutoff). Results from FPLC chromatography suggest that this enzyme may be associated with larger protein complexes, one of the components probably being serum albumine.

## Conclusion

Our PDE 7A1 data and the heat-sensitive cCMP-degrading activity detected in bovine serum samples demonstrate that cCMP PDEs really exist. We plan to characterize PDE 7A in more detail in enzyme kinetics experiments using fluorescent cNMP analogues. Moreover, we plan to purify and concentrate the cCMP-degrading activity in bovine serum in order to further characterize it with various substrates and inhibitors. In addition, we will also test organ extracts, specifically from liver (mouse and pig) in order to confirm the results previously reported in the literature and to finally reveal the identity of the “cCMP-specific” and “multifunctional” cCMP-PDE.
